# Three-dimensional facial features of suicide risk in females with depression

**DOI:** 10.3389/fpsyt.2025.1650104

**Published:** 2026-01-22

**Authors:** Jie Yang, Lingui Chen, Ling Zhang, Jing-Dong J. Han, Gang Wang

**Affiliations:** 1 Beijing Key Laboratory of Mental Disorders, National Clinical Research Center for Mental Disorders & National Center for Mental Disorders, Beijing Anding Hospital, Capital Medical University, Beijing, China; 2 Advanced Innovation Center for Human Brain Protection, Capital Medical University, Beijing, China; 3 National Institute of Biological Science Joint Graduate Program, Peking University, Beijing, China; 4 National Institute of Biological Science Joint Graduate Program, Tsinghua University, Beijing, China; 5 Peking-Tsinghua Center for Life Sciences, Academy for Advanced Interdisciplinary Studies, Center for Quantitative Biology (CQB), Peking University, Beijing, China

**Keywords:** major depressive disorder, suicide risk, 3D facial features, 3D imaging, female, sex-specific

## Abstract

**Aim:**

Suicide is the most severe consequence of Major Depressive Disorder (MDD). Current risk assessments rely heavily on subjective self-reports, which lack reliability. Emerging technologies, such as facial and behavioral recognition devices, are being explored to improve suicide risk evaluation. This study aimed to examine the potential of 3D facial features in identifying suicide risk and uncovering sex-specific characteristics in patients with MDD.

**Methods:**

We conducted a cross-sectional study involving 222 MDD patients. Suicide-related information was collected from caregivers, while independent raters assessed depressive symptoms and recorded sociodemographic data. Three-dimensional facial scans were acquired using the 3dMDface System, followed by preprocessing to extract key facial landmarks. Sex-stratified subgroup analyses were performed to identify suicide risk-associated facial features. Logistic regression analysis was used to evaluate predictors, including demographic data, clinical characteristics, and the identified facial markers.

**Results:**

Data from 203 patients were analyzed, including 110 in the suicide-risk group and 93 in the non-suicidal group. The suicidal group exhibited significantly shorter philtrum length (t = 2.137, *p* < 0.05). Analyses revealed sex-specific facial patterns, with males demonstrating suicide risk association with philtrum depth (t=2.389, *p* < 0.05) and females showing nose-eye distance variations (U = 1121, *p* < 0.05). Logistic regression identified female (OR = 2.055, 95% CI: 1.107-3.873, *p* < 0.05) and shallow philtrum (OR = 0.644, 95% CI: 0.419-0.952, *p* < 0.05) as potential factors, with a significant interaction effect (OR = 1.963, 95% CI: 0.419-0.952, *p* < 0.05).

**Conclusion:**

This study identified sex-specific facial features associated with suicide risk in MDD, with reduced philtrum depth in females emerging as a correlate. These objective measures could complement current clinical risk assessments, though further longitudinal validation is required.

**Clinical trial registration:**

https://www.chictr.org.cn, identifier ChiCTR2400090458.

## 1 Introduction

Major Depressive Disorder (MDD) poses a profound and growing public health challenge, with its global incidence having risen by approximately 50% over the past three decades (1, 2). By 2021, depressive disorders ranked as the 12th leading cause of years lived with disability worldwide (3), underscoring the urgent need for effective intervention strategies. Among the most severe consequences of MDD is suicide. Although suicidal ideation and behaviors are transdiagnostic phenomena (4), MDD remains their strongest predictor (5). Supporting this, Lucht et al. (6) demonstrated that negative mood states significantly predict both the escalation and persistence of suicidal thoughts. The scale of the issue is further highlighted by a meta-analysis in China, which found that 53.1% of MDD patients experienced suicidal ideation, 17.5% made plans, and 23.7% attempted suicide at some point in their lives (7). Developmental evidence confirms adolescence as a high-risk period for suicidal behavior. In a UK cohort, 12% of 16-year-olds with suicidal ideation attempted suicide within five years (8), consistent with epidemiological data showing peak onset of suicidality occurs between ages 11-17 (9).

This high prevalence necessitates improved methods for the early identification of suicidality. Current clinical practice relies heavily on self-report instruments, such as the Beck Scale for Suicide Ideation (10) and the Columbia-Suicide Severity Rating Scale (11). While invaluable, these tools share a critical limitation due to their dependence on patients’ subjective recall and willingness to disclose, potentially resulting in unintentional inaccuracy or deliberate concealment. Given the established link between suicide risk and depression severity (5), the search for objective biomarkers has gained momentum. Emerging technologies that analyze facial expressions, which are a known indicator of depressive states, offer a promising avenue to supplement traditional assessments (12). These integrated systems use video-based behavioral analysis for multidimensional evaluation (13–15). For instance, researchers have observed that individuals at high suicide risk often exhibit gaze aversion when questioned about suicide, a behavior that is strongly correlated with suicidal ideation (16, 17). Other subtle cues, such as reduced blinking and decreased eyelid movement, have also been noted (18). The evidence regarding specific facial expressions, however, is mixed. While reduced smiling has been associated with suicidal thoughts (16, 19), one recent study intriguingly found that expressions of disgust might be even more accurate indicators of risk than smiles (20). These inconsistent findings across the literature may stem from methodological constraints, as most existing systems rely on standard two-dimensional (2D) imaging, which captures only surface-level details and lacks the depth information necessary to detect nuanced facial dynamics.

This limitation highlights the potential of three-dimensional (3D) facial imaging, a technology that captures the face’s detailed geometry through x, y, and z coordinates. Unlike 2D photographs, 3D scans generate comprehensive facial models in seconds, enabling precise measurement of subtle morphological traits. In practice, the reliability and efficiency of 3D imaging contribute to its emerging role as a valuable tool in medicine (21–24). Crucially, by quantifying depth and angular relationships, 3D technology permits rigorous symmetry analysis and other detailed assessments that are simply not possible with 2D images (25). The precision of 3D imaging, now applied in psychiatric research, enables the identification of distinct facial phenotypes. In a key study, Haque et al. (26) developed a combined 3D facial-vocal model that detected depression with 83.3% sensitivity and 82.6% specificity, demonstrating the clinical feasibility of this approach for objective mental health assessment.

Despite these technological advances, it is still an open question whether 3D facial features can distinguish suicide risk in MDD. In this initial exploration, we analyzed a cohort of patients stratified by suicide risk level, looking specifically for potential sex-specific markers. By building models that combine multiple data types, we hope to identify any associations. Finding such features would mark a first, crucial advance in developing objective assessment methods.

## 2 Methods

This analysis is derived from a cross-sectional study investigating facial features and oral microbiota in patients with MDD. The parent study was registered with the Chinese Clinical Trial Registry (ChiCTR-2400090458). We recruited participants diagnosed with MDD from the inpatient unit at Beijing Anding Hospital’s Depression Treatment Center. Trained examiners collected 3D facial imaging data using standardized protocols. All participants completed comprehensive assessments of affective symptoms.

### 2.1 Participants

The participants were recruited from Beijing Anding Hospital and assessed by inpatient physicians. Using a consecutive enrollment approach, we included patients from April 2022 through December 2023. Eligibility was contingent upon the following criteria: (1) Patients diagnosed with MDD according to the International Statistical Classification of Diseases and Related Health Problems, Tenth Revision (F32-F33) (27); (2) Han Chinese descent, aged between 16 to 50 years. The exclusion criteria encompassed (1) Young Mania Rating Scale (YMRS) score > 6 points; (2) a history of craniofacial trauma or surgery. All participants gave written informed consent. Standard care comprised antidepressants, with antipsychotics or mood stabilizers added based on clinical assessment.

The study received approval from the Ethics Committee of Beijing Anding Hospital, Capital Medical University (Approval number: 2021-Science Research Program-104). Participants were not paid for their involvement, and all provided written informed consent before participating.

### 2.2 Assessment

#### 2.2.1 Suicide-related assessment

To reduce the risk of patients concealing information, we collected suicide-related data from medical records containing both patient and caregiver reports. Participants with prior suicide attempts, self-harm, or current suicidal ideation were classified as suicide risk (28). This classification aligns with clinical psychiatric practice for suicide risk assessment. Data were extracted directly from medical records to minimize underreporting bias (29).

#### 2.2.2 Affective symptom assessment

1. 17-item Hamilton Depression Rating Scale (HDRS-17), a clinician-administered scale with items scored 0-4 (total score range: 0-68) (30). We used the validated Chinese version (31).
2. 9-item Patient Health Questionnaire (PHQ-9), a self-report measure with items scored 0-3 (total score range: 0-27) (32). The Chinese version has demonstrated validity (33).
3. Hamilton Anxiety Scale (HAMA): Clinician-administered 14-item scale (0–4 per item; total score range: 0-56), widely used in depression research (34, 35).
4. Generalized Anxiety Disorder-7 (GAD-7): Validated 7-item self-report measure (0–3 per item; total score range: 0-21) for core anxiety symptoms (36).
5. Young Mania Rating Scale (YMRS): Clinician-administered scale assessing manic symptoms across 11 items. Each item is scored from 0 to 4 (items 5, 6, and 8 scored 0-8), yielding a total score range of 0-60 (37). We used the YMRS to exclude participants with manic or hypomanic features.

#### 2.2.3 Momentary mood assessment

Immediate Mood Scale (IMS), a 22-item self-report measure, assessed transient affective states using a validated Chinese version with a 7-point Likert scale (-3 [hopeless] to +3 [hopeful]) (38). Total scores were calculated by summing all items. Participants completed the IMS immediately before undergoing 3D facial imaging to control for the acute effects of mood on facial morphology.

### 2.3 3D facial features acquisition and processing

**Figure 1:**
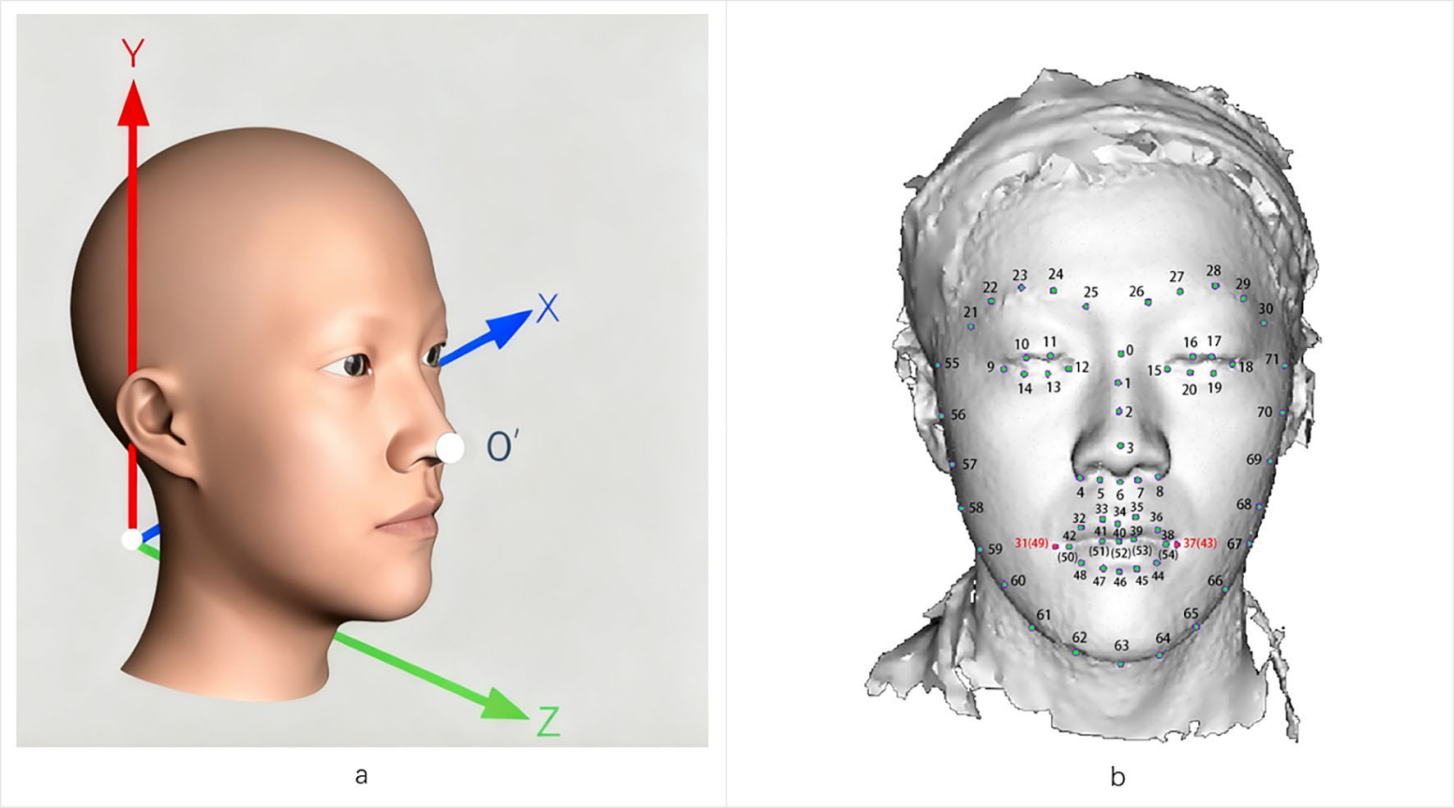
3D facial landmarks are labeled as the basis for morphological quantitative analysis. **(a)** Representation of a 3D facial image based on meshes within a three-dimensional coordinate space. The face exhibits overall symmetry concerning the X-Y-Z plane. **(b)** Example of 72 landmarks on a 3D facial surface.

We acquired 3D facial images using the 3dMDface System (www.3dmd.com) under standardized lighting conditions (natural or artificial). Participants removed facial accessories to ensure optimal capture quality. The system’s cameras captured images from fixed angles, with proprietary algorithms generating raw 3D data (0.2mm accuracy), including point clouds and texture files. The preprocessing pipeline involved identifying and aligning key facial landmarks, with pose normalization performed through global rotation of the complete facial model centered at the nasal tip (designated as point O’). This rotation step preserved the original point cloud distribution without altering individual morphological characteristics. Subsequent alignment was conducted using multiple landmarks. To reduce noise and facilitate group-level comparisons, surface smoothing was applied using the validated MeshMonk (39), which generates landmark-derived facial features that closely correspond to anatomical measurements. Collectively, these steps standardized the dataset for robust three-dimensional morphometric analysis. **Figure 1A** shows the 3D facial reconstruction with XYZ axis orientation for spatial analysis.2.5 Face landmarks labeling. Each sample contained 72 standardized facial landmarks (P_0_-P_71_; **Figure 1B**) encompassing key morphological features: facial contours, oral commissures, nasal alae, and ocular canthi. All directional references adopt the observer’s perspective unless specified.

From pre-annotated landmarks, we selected 47 features based on their relevance to psychiatric research. Analyses comprised linear, angular, and surface measurements, enhanced by ratio and midpoint techniques. The photographer-recorded laterality was converted to anatomical orientation. Additional details are provided in the **Supplementary Materials**.

### 2.4 Statistical analysis

All statistical analyses were conducted using R software, version 4.3.1. Categorical variables were expressed in terms of frequency and percentage, with intergroup comparisons made using the chi-square (χ2) test. For continuous variables, the mean ± standard deviation (SD) was reported following a Shapiro-Wilk test for normality. Normally distributed variables were analyzed using the independent samples t-test, while the non-parametric Mann-Whitney U test was applied for those not meeting normality criteria. Logistic regression was used to identify clinical and facial variables correlated with suicide risk. To address the issue of multiple comparisons arising from testing numerous facial features against suicide risk, we controlled the false discovery rate using the Benjamini-Hochberg procedure. Statistical significance was set at *p* < 0.05, indicating a meaningful difference.

## 3 Results

We screened 222 participants and excluded 19 (10 withdrew consent, 9 had poor-quality 3D images), leaving 203 for analysis. These patients were divided into two groups based on suicide risk: the suicide risk group (SR, n = 110) and the no-suicide risk group (NSR, n = 93).

### 3.1 Intergroup comparison of demographic, clinical characteristics, and 3D facial features

Demographic and clinical characteristics were largely comparable between the SR and NSR groups. No significant differences were observed in age, body mass index, diagnostic subtype, presence of psychotic symptoms, or scores on depression and anxiety scales. The SR included a significantly higher proportion of female patients (62%) compared to the NSR group (46%; χ² = 4.329, *p* < 0.05). Detailed demographic and clinical data are provided in **Table 1**.

**Table 1:** Characteristics of patients with MDD in the NSR and SR.

Variables	Overall (n=203)	NSR (n=93)	SR (n=110)	*U*/χ2	*p*
	Mean (SD)	Mean (SD)	Mean (SD)		
Age	26.95 (9.96)	27.87 (10.47)	26.17 (9.49)	5333.5	0.600
Gender, n (%)				4.329	0.038^*^
Male	92 (45%)	50 (54%)	42 (38%)		
Female	111 (55%)	43 (46%)	68 (62%)		
BMI	23.25 (4.66)	23.74 (4.89)	22.83 (4.45)	5580.0	0.265
Diagnosis, n (%)				0.090	0.765
Single-episode depressive disorder	106 (52%)	47 (51%)	59 (54%)		
Recurrent depressive disorder	97 (48%)	46 (49%)	51 (46%)		
Psychotic symptoms, n (%)				0.025	0.873
Without Psychotic symptoms	183 (90%)	83 (89%)	100 (91%)		
With Psychotic symptoms	20 (10%)	10 (11%)	10 (9%)		
IMS	-1.22 (27.44)	3.23 (27.66)	-4.99 (26.81)	5934.0	0.050
PHQ-9	15.30 (6.48)	14.48 (6.32)	15.98 (6.56)	4416.0	0.094
GAD-7	10.27 (5.74)	10.62 (5.72)	9.96 (5.77)	5484.5	0.376
HDRS-17	20.51 (7.52)	20.00 (7.45)	20.95 (7.58)	4732.0	0.359
HAMA	18.76 (9.14)	19.76 (9.39)	17.92 (8.87)	5717.0	0.149

NSR, No-suicide risk group; SR, Suicide risk group; BMI, Body mass index; IMS, Immediate Mood Scale; PHQ-9, 9-item Patient Health Questionnaire; GAD-7, Generalized Anxiety Disorder; HDRS-17, 17-item Hamilton Depression Rating Scale; HAMA, Hamilton Anxiety Scale^; *^p < 0.05.

Among the 47 facial features analyzed, philtrum length was the only measure that differed significantly between groups. Patients in the SR group had a shorter philtrum length than those in the NSR group (t = 2.137, *p* < 0.05). No other 3D facial features showed statistically significant differences in the overall sample (**Table 2**).

**Table 2:** Differences in 3D facial features between NSR and SR.

Variables	NSR (n=93)	SR (n=110)	t/*U*	*p*
	Mean (SD)	Mean (SD)		
Distance features (mm)				
Forehead width	122.44 (6.64)	122.30 (5.80)	0.164	0.870
Face width	155.00 (9.09)	153.18 (8.17)	1.486	0.139
Face length	132.68 (8.17)	131.86 (6.83)	0.772	0.441
Jaw width	139.56 (10.77)	135.95 (9.30)	1.832	0.069
Nose width	28.76 (2.23)	28.38 (2.30)	1.214	0.226
Nose height	14.49 (1.80)	14.47 (1.74)	5200	0.839
Nose depth	17.53 (2.74)	17.39 (2.59)	0.360	0.719
Nose length	41.36 (3.25)	41.72 (2.79)	-0.833	0.406
Part nose x	28.69 (2.25)	28.29 (2.27)	1.258	0.210
Part nose y	50.91 (3.17)	51.05 (2.73)	-0.331	0.741
Philtrum depth	3.83 (1.75)	3.57 (1.58)	1.101	0.273
Philtrum length	16.14 (2.45)	15.45 (2.13)	2.137	0.034*
Philtrum length flat	15.56 (2.44)	14.91 (2.28)	1.964	0.051
Mouth width	49.38 (4.27)	48.38 (3.89)	5854	0.077
Part mouth x	49.33 (4.25)	48.34 (3.88)	1.712	0.089
Dist nose lip	22.69 (2.40)	22.34 (2.21)	1.099	0.273
Upper lip thick	9.52 (1.64)	9.63 (1.62)	-0.479	0.633
Lower lip thick	10.90 (1.71)	11.20 (1.67)	-1.257	0.210
Chin depth	24.32 (17.58)	25.30 (19.88)	5208	0.824
Chin length	41.53 (13.73)	41.72 (15.79)	5323	0.619
center eye width	25.93 (1.55)	25.93 (1.52)	0.002	0.998
Right eye width	25.77 (1.64)	25.94 (1.48)	-0.774	0.440
Dist inner eyes	39.88 (3.01)	40.08 (2.79)	-0.493	0.623
Dist outer eyes	90.30 (5.11)	90.66 (4.24)	-0.536	0.592
Dist Leye nose	19.61 (2.04)	19.91 (1.62)	4567	0.189
Dist Reye nose	20.20 (1.60)	20.14 (1.65)	0.286	0.775
Part Leye x	25.29 (1.47)	25.31 (1.37)	-0.119	0.905
Part Leye y	7.64 (1.85)	7.39 (1.84)	-0.604	0.547
Part Reye x	25.17 (1.52)	25.27 (1.39)	-0.473	0.637
Part Reye y	7.62 (1.77)	7.88 (1.79)	-1.067	0.287
Part Leyepit y	28.77 (3.05)	28.58 (3.07)	0.430	0.667
Part Reyepit y	28.73 (2.79)	28.82 (3.17)	4954	0.700
Angle features (°)				
Philtrum slope x	1.52 (0.04)	1.53 (0.03)	4440	0.106
Philtrum slope y	0.25 (0.10)	0.25 (0.11)	0.284	0.777
Philtrum slope z	1.33 (0.11)	1.33 (0.11)	-0.068	0.946
Chin slope x	1.53 (0.04)	1.53 (0.04)	5351	0.572
Chin slope y	0.60 (0.25)	0.61 (0.26)	5201	0.838
Chin slope z	0.98 (0.25)	0.96 (0.26)	4567	0.189
center eye slope x	0.21 (0.08)	0.21 (0.06)	4832	0.498
center eye slope y	1.48 (0.05)	1.47 (0.05)	1.017	0.310
center eye slope z	1.40 (0.09)	1.40 (0.07)	5335	0.599
Right eye slope x	0.21 (0.07)	0.22 (0.06)	-1.752	0.081
Right eye slope y	1.47 (0.05)	1.46 (0.05)	5462	0.406
Right eye slope z	1.41 (0.07)	1.39 (0.07)	1.578	0.116
Area features (mm^2^)				
center eye area	126.40 (30.97)	129.19 (30.98)	-0.639	0.524
Right eye area	130.40 (31.82)	132.07 (33.01)	-0.366	0.715
Midpoint projection (mm)				
Forehead height	120.04 (4.38)	120.40 (4.40)	-0.575	0.566

NSR, No-suicide risk group; SR, Suicide risk group; ^*^p < 0.05.

### 3.2 Sex-specific 3D facial features for suicide risk

When analyses were stratified by sex, distinct facial patterns emerged. Among male patients, a deeper philtrum was significantly associated with suicide risk (t = 2.389, *p* < 0.05). In female patients, a shorter distance between the nose and the left eye (Dist Leye nose) showed a stronger association with suicide risk (U = 1121, *p* < 0.05). The complete set of sex-stratified analyses is presented in **Table 3**.

**Table 3:** Differences in 3D facial features between NSR and SR by gender.

Variables	Male NSR (n=50) vs. SR (n =42)		Female NSR (n=43) vs. SR (n =68)	
	t/*U*	*p*	t/*U*	*p*
Distance features (mm)				
Forehead width	-0.080	0.937	-0.977	0.331
Face width	0.828	0.410	0.131	0.896
Face length	1.540	0.127	-1.362	0.176
Jaw width	1.396	0.166	0.001	0.999
Nose width	1.157	0.251	-0.904	0.368
Nose height	970	0.533	1430	0.849
Nose depth	-0.360	0.720	0.146	0.884
Nose length	-1.308	0.194	-1.046	0.299
Part nose x	1.232	0.221	-0.886	0.378
Part nose y	-0.609	0.544	-0.938	0.351
Philtrum depth	2.389	0.019 *	-1.112	0.269
Philtrum length	1.799	0.075	0.418	0.677
Philtrum length flat	1.400	0.165	0.600	0.550
Mouth width	1217	0.192	1493	0.854
Part mouth x	1.449	0.151	0.133	0.894
Dist nose lip	0.730	0.467	0.067	0.947
Upper lip thick	-0.507	0.614	-0.193	0.848
Lower lip thick	-0.439	0.662	-1.614	0.110
Chin depth	1130	0.533	1393	0.678
Chin length	1174	0.333	1380	0.622
center eye width	0.067	0.946	-0.227	0.821
Right eye width	-0.228	0.820	-1.219	0.226
Dist inner eyes	-0.603	0.548	-1.196	0.234
Dist outer eyes	-0.432	0.667	-1.231	0.222
Dist Leye nose	971	0.538	1121	0.039 *
Dist Reye nose	0.022	0.983	-0.319	0.750
Part Leye x	-0.125	0.901	-0.240	0.811
Part Leye y	0.115	0.908	0.001	0.999
Part Reye x	0.043	0.966	-1.208	0.230
Part Reye y	0.206	0.838	-0.868	0.388
Part Leyepit y	1.385	0.170	-0.892	0.375
Part Reyepit y	1166	0.365	1234	0.168
Angle features (°)				
Philtrum slope x	891	0.214	1297	0.319
Philtrum slope y	1.678	0.097	-1.166	0.247
Philtrum slope z	1.163	0.248	0.787	0.434
Chin slope x	1022	0.829	1540	0.639
Chin slope y	1041	0.947	1436	0.877
Chin slope z	1063	0.922	1492	0.858
center eye slope x	941	0.395	1508	0.783
center eye slope y	0.484	0.630	0.344	0.732
center eye slope z	1143	0.468	1430	0.849
Right eye slope x	-1.389	0.168	-1.389	0.168
Right eye slope y	1118	0.597	1428	0.839
Right eye slope z	1.163	0.248	0.787	0.434
Area features (mm^2^)				
center eye area	0.634	0.528	-0.907	0.367
Right eye area	0.416	0.678	-0.012	0.991
Midpoint projection (mm)				
Forehead height	0.212	0.833	-1.092	0.278

NSR, No-suicide risk group; SR, Suicide risk group; ^*^p < 0.05.

### 3.3 Exploratory factors associated with suicide risk in MDD

An exploratory logistic regression was performed to evaluate the joint contribution of sex and facial morphology to suicide risk. The results indicated that female sex (OR = 1.944, 95% CI: 1.107–3.873) and shallower philtrum depth (OR = 0.650, 95% CI: 0.419–0.952) were each independently associated with suicide risk (both *p* < 0.05). A significant interaction was observed between female sex and philtrum depth (OR = 2.349, 95% CI: 1.079–3.655, *p* < 0.05), suggesting a combined effect on suicide risk. After adjusting for multiple comparisons using the Benjamini-Hochberg procedure, the association for philtrum depth and the interaction term remained marginally significant (*p*_adj = 0.068). Full regression results are shown in **Table 4**.

**Table 4:** Logistic regression of suicide risk by sex and facial features.

Variables	Estimate	OR	95%CI	*p*	*p_adj^1^*
Gender (Female/Male)	0.720	2.055	1.107-3.873	0.024*	0.068
Philtrum length	-0.260	0.771	0.560-1.050	0.103	0.154
Philtrum depth	-0.441	0.644	0.419-0.952	0.034*	0.068
Dis Leye nose	0.247	1.280	0.868-1.949	0.217	0.260
Female* Philtrum depth	0.674	1.963	1.079-3.655	0.030*	0.068
Female* Dis Leye nose	0.230	1.258	0.671-2.364	0.470	0.470

^1^P-values were corrected using the Benjamini-Hochberg method; *p < 0.05.

## 4 Discussion

Our study identified distinct facial morphology patterns associated with suicide risk in depression, with variations between sexes. Reduced philtrum depth showed a particular association with suicide risk in women, supported by a significant interaction term between female sex and philtrum morphology. While these findings should be interpreted as exploratory, they contribute to the growing interest in developing objective markers for suicide risk assessment.

Women consistently show higher rates of suicidal ideation and attempts (40, 41), which aligns with the sex-specific associations observed in our study. Neurochemical profiles reveal that women typically show higher densities of 5-Hydroxytryptamine receptor 1A (5-HT_1A_) and lower serotonin transporter (5-HTT) availability in key emotional processing regions like the hippocampus (42). This pattern may contribute to their increased vulnerability to stress and depression (43, 44). Consistent evidence from Arango et al. (45) and Aleksandra et al. (46) demonstrated reduced 5-HT_1A_ receptor and 5-HTT function across multiple brain regions, including the dorsal raphe nucleus, prefrontal cortex, and hypothalamus, in depressed individuals who died by suicide, indicating that serotonergic dysfunction is a key neurobiological basis of suicide. Furthermore, estrogen fluctuations during pregnancy and menopause in females can modulate serotonin activity and serotonin transporter availability (47, 48). From a developmental perspective, the link between philtrum morphology and suicide risk in women may share embryological origins. During weeks 4–7 of gestation, neural crest cells give rise to both the midfacial prominences forming the philtrum and the limbic structures regulating emotional processing (49, 50). Lower testosterone levels in female embryos may be associated with shallower philtrum development (51) and concurrently influence the maturation of emotion-regulation pathways such as the prefrontal-amygdala circuit (52). It remains unclear whether a shallower philtrum arises primarily from female sex characteristics or reflects atypical development of emotional neurocircuitry. Future studies should focus on high-risk female populations to examine how philtrum depth correlates with emotional regulation function and suicide vulnerability.

Facial features capture both biological traits and emotional expression. Muscle movements around the mouth directly alter philtrum morphology. For instance, contraction of the orbicularis oris shortens the upper lip and flattens the philtrum, an expression linked to anxiety (53). Similarly, activation of the depressor anguli oris elongates and shallowens the philtrum in sad or fearful expressions (54). These expressive patterns are consistent across cultures (55). Our findings reveal that a shallower philtrum depth remains detectable even at rest and is significantly associated with suicide risk, suggesting that emotion-related facial movements may leave stable morphological traces or reflect early neurodevelopmental shaping of facial structures. Supporting evidence comes from studies of Chinese youth linking resting angry or anxious expressions to suicide risk (20). Most notably, the combination of reduced philtrum depth and female sex shows an association with suicide risk (OR = 1.963, *p* < 0.05), further suggesting potential neurodevelopmental interactions. The persistence of these morphological markers across emotional states underscores their value as indicators of vulnerability, complementing traditional behavioral assessments in clinical risk evaluation.

Conventional methods relying on 2D images or videos remain constrained by sensitivity to lighting, pose, and other environmental variables. Our approach leverages 3D facial geometry to overcome these limitations, aligning with the growing interest in more robust and physiologically meaningful features. This direction is supported by a number of recent methodological innovations, which also highlight the potential advantages of integrating 3D data. Wang et al. (56) developed a sophisticated multimodal spatiotemporal feature set to capture depression-related manifestations; their framework’s reliance on 2D video leaves it susceptible to lighting and pose variations. The incorporation of 3D features could directly mitigate these challenges, simplifying data normalization and enhancing model generalization. The behavioral primitives proposed by Song et al. (57) for spectral analysis could be more accurately defined using 3D facial data, as the geometry provides a direct correlate of muscle activity, free from appearance-based artifacts. The value of 3D integration is further evident in architectures like the Depression Multi-view Graph Neural Network by Wu et al. (58), where anatomically grounded spatial coordinates from 3D scans would provide a more physically accurate and interpretable basis for modeling interactions between facial regions. Beyond enhancing existing models, 3D features are particularly promising for identifying cross-modal depression characteristics in real-world contexts. The multimodal corpus contributed by Zou et al. (59), derived from semi-structured interviews, underscores the importance of nuanced behavioral cues. 3D facial quantification is ideally suited to this task, enabling precise measurement of subtle dynamics often lost in 2D representations. This capability supports a richer quantitative analysis of non-verbal behavior. These efforts reflect a growing interest in multimodal datasets (60) that incorporate 3D facial data for finer-grained behavioral measurement.

From a methodological perspective, we acknowledge several considerations regarding feature stability. Our preprocessing pipeline, while necessary for standardization, may affect subtle morphological variations. The global rotation around the nasal tip preserves overall point cloud distribution but could obscure orientation-dependent shape details. Similarly, surface smoothing with MeshMonk (39), though effective for noise reduction, might attenuate fine-grained morphological information. These technical factors, combined with the moderate sample size and marginal significance after multiple testing correction, highlight the preliminary nature of our findings. Future studies focusing on highly localized facial features may benefit from alternative registration strategies or multi-scale analytical approaches that better balance noise reduction with feature preservation.

In terms of clinical interpretation, it is important to clarify the temporal relationship between risk assessment and data collection. Suicide risk was determined based on historical medical records, including prior suicide attempts or self-harm episodes, while 3D facial imaging was conducted at a subsequent time point. Therefore, the observed associations reflect retrospective correlations rather than predictive validity. The results suggest that certain facial morphological patterns may persist as stable markers of vulnerability even during non-acute phases among individuals with a history of suicidality. However, the study design does not support the use of these features to predict future suicidal events. Rather, these findings highlight the potential of facial morphology as an enduring indicator of underlying vulnerability, which may complement dynamic risk assessments in future longitudinal studies.

This study has several limitations that should be considered. First, our feature selection was based mainly on univariate differential testing. While this approach detects features with significant group mean differences, it cannot adjust for multicollinearity or capture complex interactive effects between multiple features and the outcome. Future studies should consider applying diverse feature selection methods for validation. Second, the sample size of 203 participants from a single source offers limited statistical power to reliably identify associations with small to moderate effect sizes, even after controlling for potential confounders. This increases the risk of unstable subgroup estimates or overlooking meaningful predictors. Finally, the cross-sectional design restricts insight into how facial morphology changes with symptom progression over time. Accordingly, these findings should be viewed as exploratory. They provide preliminary clues and generate hypotheses for future mechanistic and modeling research, but their robustness and clinical relevance need to be confirmed through larger, prospective, and multi-center studies.

## 5 Conclusion

This study provides preliminary evidence that 3D facial morphology, particularly philtrum depth, may serve as a sex-specific marker associated with suicide risk in women with major depressive disorder. While these results highlight the potential of objective facial metrics to complement current risk assessment tools, the moderate sample size and cross-sectional design underscore the need for further validation. Future longitudinal and multi-center studies are essential to confirm the stability and predictive value of these facial features, and to explore their integration into clinically actionable frameworks for suicide prevention.

## Data Availability

The datasets presented in this article are not readily available because the datasets generated during this study are not publicly available due to participant privacy concerns and institutional regulations. Requests to access the datasets should be directed to gangwangdoc@ccmu.edu.cn.

## References

[1] Monroe SM Harkness KL . Major depression and its recurrences: life course matters. Annu Rev Clin Psychol. (2022) 18:329–57. doi: 10.1146/annurev-clinpsy-072220-021440, PMID: 35216520

[2] Liu Q He H Yang J Feng X Zhao F Lyu J . Changes in the global burden of depression from 1990 to 2017: Findings from the Global Burden of Disease study. J Psychiatr Res. (2020) 126:134–40. doi: 10.1016/j.jpsychires.2019.08.002, PMID: 31439359

[3] GBD 2021 Dis Injuries Collaborators . Global incidence, prevalence, years lived with disability (YLDs), disability-adjusted life-years (DALYs), and healthy life expectancy (HALE) for 371 diseases and injuries in 204 countries and territories and 811 subnational locations, 1990-2021: A systematic analysis for the Global Burden of Disease Study 2021. Lancet. (2024) 403(10440):2133–61. doi: 10.1016/S0140-6736(24)00757-8, PMID: 38642570 PMC11122111

[4] Oquendo MA Baca-García E Mann JJ Giner J . Issues for DSM-V: suicidal behavior as a separate diagnosis on a separate axis. Am J Psychiatry. (2008) 165:1383–4. doi: 10.1176/appi.ajp.2008.08020281, PMID: 18981069 PMC3776420

[5] Ribeiro JD Huang X Fox KR Franklin JC . Depression and hopelessness as risk factors for suicide ideation, attempts and death: meta-analysis of longitudinal studies. Br J psychiatry: J Ment science. (2018) 212:279–86. doi: 10.1192/bjp.2018.27, PMID: 29587888

[6] Lucht L Spangenberg L Forkmann T Hallensleben N Rath D Strauss M . Association of real-time assessed mood, affect and suicidal ideation in psychiatric inpatients with unipolar depression. Clin Psychol Psychother. (2022) 29:1580–6. doi: 10.1002/cpp.2741, PMID: 35383387

[7] Lin J-Y Huang Y Su Y-A Yu X Lyu X-Z Liu Q . Association between perceived stressfulness of stressful life events and the suicidal risk in chinese patients with major depressive disorder. Chin Med J. (2018) 131:912–9. doi: 10.4103/0366-6999.229898, PMID: 29664050 PMC5912056

[8] Mars B Heron J Klonsky ED Moran P O’Connor RC Tilling K . Predictors of future suicide attempt among adolescents with suicidal thoughts or non-suicidal self-harm: a population-based birth cohort study. Lancet Psychiatry. (2019) 6:327–37. doi: 10.1016/s2215-0366(19)30030-6, PMID: 30879972 PMC6494973

[9] Hutchinson E Scott L Choukas-Bradley S Silk J . Interpersonal risk factors for suicide in daily life among young people: A review of intensive longitudinal studies. Dev Psychopathol. (2025) 37:2196–216. doi: 10.1017/s0954579424001810, PMID: 39743871

[10] Beck AT Kovacs M Weissman A . Assessment of suicidal intention: the Scale for Suicide Ideation. J Consult Clin Psychol. (1979) 47:343–52. doi: 10.1037//0022-006x.47.2.343, PMID: 469082

[11] Posner K Brown GK Stanley B Brent DA Yershova KV Oquendo MA . The Columbia-Suicide Severity Rating Scale: initial validity and internal consistency findings from three multisite studies with adolescents and adults. Am J Psychiatry. (2011) 168:1266–77. doi: 10.1176/appi.ajp.2011.10111704, PMID: 22193671 PMC3893686

[12] Hu B Tao Y Yang M . Detecting depression based on facial cues elicited by emotional stimuli in video. Comput Biol Med. (2023) 165:107457. doi: 10.1016/j.compbiomed.2023.107457, PMID: 37708718

[13] Liu D Liu B Lin T Liu G Yang G Qi D . Measuring depression severity based on facial expression and body movement using deep convolutional neural network. Front Psychiatry. (2022) 13:1017064. doi: 10.3389/fpsyt.2022.1017064, PMID: 36620657 PMC9810804

[14] Hunter L Roland L Ferozpuri A . Emotional expression processing and depressive symptomatology: eye-tracking reveals differential importance of lower and middle facial areas of interest. Depression Res Treat. (2020) 2020:1049851. doi: 10.1155/2020/1049851, PMID: 32395340 PMC7199636

[15] Galatzer-Levy I Abbas A Ries A Homan S Sels L Koesmahargyo V . Validation of visual and auditory digital markers of suicidality in acutely suicidal psychiatric inpatients: proof-of-concept study. J Med Internet Res. (2021) 23:e25199. doi: 10.2196/25199, PMID: 34081022 PMC8212625

[16] Eigbe N Baltrušaitis T Morency L-p Pestian JP . “ Toward Visual Behavior Markers of Suicidal Ideation,” In: M2018 13th IEEE International Conference on Automatic Face & Gesture Recognition (FG 2018), Xi'an, China, (2018) 530–4. doi: 10.1109/FG.2018.00085

[17] Shah A Sharma V Vaibhav V Alismail M Morency L . . “Multimodal Behavioral Markers Exploring Suicidal Intent in Social Media Videos.” In: 2019 International Conference on Multimodal Interaction. (2019).

[18] Liu S Lu C Alghowinem S Gotoh L Breazeal C Park HW . “ Explainable AI for Suicide Risk Assessment Using Eye Activities and Head Gestures”. In: Degen H, Ntoa S, editors. Artificial Intelligence in HCI. HCII 2022. Lecture Notes in Computer Science, Cham: Springer. (2022) 13336. doi: 10.1007/978-3-031-05643-7_11

[19] Laksana E Baltrusaitis T Morency LP Pestian JP . (2017). Investigating facial behavior indicators of suicidal ideation, in: IEEE International Conference on Automatic Face & Gesture Recognition, .

[20] Hu CS Zhang H Short LA Hu S . Individuals with higher suicide risk showed more anger and disgust during rest. Death Stud. (2024) 48:9–15. doi: 10.1080/07481187.2023.2186537, PMID: 36906516

[21] Dolci C Sansone VA Gibelli D Cappella A Sforza C . Distinctive facial features in Andersen-Tawil syndrome: A three-dimensional stereophotogrammetric analysis. Am J Med Genet A. (2021) 185:781–9. doi: 10.1002/ajmg.a.62040, PMID: 33369085

[22] Meng T Guo X Lian W Deng K Gao L Wang Z . Identifying facial features and predicting patients of acromegaly using three-dimensional imaging techniques and machine learning. Front Endocrinol (Lausanne). (2020) 11:492. doi: 10.3389/fendo.2020.00492, PMID: 32849283 PMC7403213

[23] Park H Min J Koh KS . Three-dimensional anthropometry for evaluating philtrum contour in patients with unilateral cleft lip: comparison between photographic assessment and 3-dimensional anthropometry. J craniofacial surgery. (2023) 34:2061–5. doi: 10.1097/scs.0000000000009667, PMID: 37622549

[24] Postema FAM Matthews H Hopman SMJ Merks JHM Suttie M Hoskens H . 3D analysis of facial morphology in Dutch children with cancer. Comput Methods Programs Biomed. (2021) 205:106093. doi: 10.1016/j.cmpb.2021.106093, PMID: 33882417

[25] Berssenbrügge P Berlin NF Kebeck G Runte C Jung S Kleinheinz J . 2D and 3D analysis methods of facial asymmetry in comparison. J cranio-maxillo-facial surgery: Off Publ Eur Assoc Cranio-Maxillo-Facial Surgery. (2014) 42:e327–34. doi: 10.1016/j.jcms.2014.01.028, PMID: 24507934

[26] Haque A Guo M Miner AS Fei-Fei Li . Measuring Depression Symptom Severity from Spoken Language and 3D Facial Expressions. ArXiv, abs/1811.08592. (2018).

[27] World Health Organization . The ICD-10 Classification of Mental and Behavioural Disorders: Diagnostic Criteria for Research. Geneva: World Health Organization. (1993).

[28] Raue PJ Ghesquiere AR Bruce ML . Suicide risk in primary care: identification and management in older adults. Curr Psychiatry Rep. (2014) 16:466. doi: 10.1007/s11920-014-0466-8, PMID: 25030971 PMC4137406

[29] Yarborough BJH Stumbo SP Schneider JL Richards JE Hooker SA Rossom RC . Patient expectations of and experiences with a suicide risk identification algorithm in clinical practice. BMC Psychiatry. (2022) 22:494. doi: 10.1186/s12888-022-04129-1, PMID: 35870919 PMC9308306

[30] Hamilton M . Development of a rating scale for primary depressive illness. Br J Soc Clin Psychol. (1967) 6:278–96. doi: 10.1111/j.2044-8260.1967.tb00530.x, PMID: 6080235

[31] Zheng YP Zhao JP Phillips M Liu JB Cai MF Sun SQ . Validity and reliability of the chinese hamilton depression rating scale. Br J psychiatry: J Ment science. (1988) 152:660–4. doi: 10.1192/bjp.152.5.660, PMID: 3167442

[32] Kroenke K Spitzer RL Williams JB . The PHQ-9: validity of a brief depression severity measure. J Gen Intern Med. (2001) 16:606–13. doi: 10.1046/j.1525-1497.2001.016009606.x, PMID: 11556941 PMC1495268

[33] Sun XY Li YX Yu CQ Li LM . Reliability and validity of depression scales of Chinese version: a systematic review. Chin J Epidemiol. (2017) 38:110–6. doi: 10.3760/cma.j.issn.0254-6450.2017.01.021, PMID: 28100388

[34] Hamilton M . The assessment of anxiety states by rating. Br J Med Psychol. (1959) 32:50–5. doi: 10.1111/j.2044-8341.1959.tb00467.x, PMID: 13638508

[35] Maier W Buller R Philipp M Heuser I . The Hamilton Anxiety Scale: reliability, validity and sensitivity to change in anxiety and depressive disorders. J Affect Disord. (1988) 14:61–8. doi: 10.1016/0165-0327(88)90072-9, PMID: 2963053

[36] Löwe B Decker O Müller S Brähler E Schellberg D Herzog W . Validation and standardization of the Generalized Anxiety Disorder Screener (GAD-7) in the general population. Med Care. (2008) 46:266–74. doi: 10.1097/MLR.0b013e318160d093, PMID: 18388841

[37] Young RC Biggs JT Ziegler VE Meyer DA . A rating scale for mania: reliability, validity and sensitivity. Br J psychiatry: J Ment science. (1978) 133:429–35. doi: 10.1192/bjp.133.5.429, PMID: 728692

[38] Nahum M Vleet TMV Sohal VS Mirzabekov JJ Rao VR Wallace DL . Immediate mood scaler: tracking symptoms of depression and anxiety using a novel mobile mood scale. JMIR mHealth uHealth. (2017) 5:e44. doi: 10.2196/mhealth.6544, PMID: 28404542 PMC5406620

[39] White JD Ortega-Castrillón A Matthews H Zaidi AA Ekrami O Snyders J . MeshMonk: Open-source large-scale intensive 3D phenotyping. Sci Rep. (2019) 9:6085. doi: 10.1038/s41598-019-42533-y, PMID: 30988365 PMC6465282

[40] Freeman A Mergl R Kohls E Székely A Gusmao R Arensman E . A cross-national study on gender differences in suicide intent. BMC Psychiatry. (2017) 17:234. doi: 10.1186/s12888-017-1398-8, PMID: 28662694 PMC5492308

[41] Nock MK Green JG Hwang I McLaughlin KA Sampson NA Zaslavsky AM . Prevalence, correlates, and treatment of lifetime suicidal behavior among adolescents: results from the National Comorbidity Survey Replication Adolescent Supplement. JAMA Psychiatry. (2013) 70:300–10. doi: 10.1001/2013.jamapsychiatry.55, PMID: 23303463 PMC3886236

[42] Jovanovic H Lundberg J Karlsson P Cerin A Saijo T Varrone A . Sex differences in the serotonin 1A receptor and serotonin transporter binding in the human brain measured by PET. Neuroimage. (2008) 39:1408–19. doi: 10.1016/j.neuroimage.2007.10.016, PMID: 18036835

[43] Zhang Z-J Wang D Man SC Ng R McAlonan GM Wong HK . Platelet 5-HT(1A) receptor correlates with major depressive disorder in drug-free patients. Prog Neuropsychopharmacol Biol Psychiatry. (2014) 53:74–9. doi: 10.1016/j.pnpbp.2014.03.004, PMID: 24657886

[44] Altieri SC Garcia-Garcia AL Leonardo ED Andrews AM . Rethinking 5-HT1A receptors: emerging modes of inhibitory feedback of relevance to emotion-related behavior. ACS Chem Neurosci. (2013) 4:72–83. doi: 10.1021/cn3002174, PMID: 23336046 PMC3547474

[45] Arango V Underwood MD Boldrini M Tamir H Kassir SA Hsiung S . Serotonin 1A receptors, serotonin transporter binding and serotonin transporter mRNA expression in the brainstem of depressed suicide victims. Neuropsychopharmacology: Off Publ Am Coll Neuropsychopharmacol. (2001) 25:892–903. doi: 10.1016/s0893-133x(01)00310-4, PMID: 11750182

[46] Wisłowska-Stanek A Kołosowska K Maciejak P . Neurobiological basis of increased risk for suicidal behaviour. Cells. (2021) 10(10):2519. doi: 10.3390/cells10102519, PMID: 34685499 PMC8534256

[47] Steiner M . Serotonin, depression, and cardiovascular disease: sex-specific issues. Acta Physiol (Oxf). (2011) 203:253–8. doi: 10.1111/j.1748-1716.2010.02236.x, PMID: 21281455

[48] Stacy M Kremer M Schulkin J . Suicide among women and the role of women’s health care providers. Obstet Gynecol Surv. (2022) 77:293–301. doi: 10.1097/ogx.0000000000001025, PMID: 35522431

[49] Roth DM Bayona F Baddam P Graf D . Craniofacial development: neural crest in molecular embryology. Head Neck Pathol. (2021) 15:1–15. doi: 10.1007/s12105-021-01301-z, PMID: 33723764 PMC8010074

[50] Cordero DR Brugmann S Chu Y Bajpai R Jame M Helms JA . Cranial neural crest cells on the move: their roles in craniofacial development. Am J Med Genet A. (2011) 155A:270–9. doi: 10.1002/ajmg.a.33702, PMID: 21271641 PMC3039913

[51] Whitehouse AJ Gilani SZ Shafait F Mian A Tan DW Maybery MT . Prenatal testosterone exposure is related to sexually dimorphic facial morphology in adulthood. Proc Biol Sci. (2015) 282:20151351. doi: 10.1098/rspb.2015.1351, PMID: 26400740 PMC4614768

[52] Lombardo MV Ashwin E Auyeung B Chakrabarti B Taylor K Hackett G . Fetal testosterone influences sexually dimorphic gray matter in the human brain. J Neurosci. (2012) 32:674–80. doi: 10.1523/jneurosci.4389-11.2012, PMID: 22238103 PMC3306238

[53] Peck SR . Atlas of Human Anatomy for the Artist (Galaxy Books). Oxford Univ Pr (2015).

[54] Ekman P Sorenson ER Friesen WV . Pan-cultural elements in facial displays of emotion. Sci (New York NY). (1969) 164:86–8. doi: 10.1126/science.164.3875.86, PMID: 5773719

[55] Harmonjones E . Handbook of cognition and emotion. Br J Psychiatry. (1999) 176:500. doi: 10.1002/0470013494

[56] Wang Y Lin Z Yang C Zhou Y Yang Y . Automatic depression recognition with an ensemble of multimodal spatio-temporal routing features. IEEE Trans Affect Computing. (2025) 16:1855–72. doi: 10.1109/TAFFC.2025.3543226

[57] Song S Jaiswal S Shen L Valstar M . Spectral representation of behaviour primitives for depression analysis. IEEE Trans Affect Computing. (2022) 13:829–44. doi: 10.1109/TAFFC.2020.2970712

[58] Wu Z Zhou L Li S Fu C Lu J Han J . DepMGNN: matrixial graph neural network for video-based automatic depression assessment. Proc AAAI Conf Artif Intell. (2025) 39:1610–9. doi: 10.1609/aaai.v39i2.32153

[59] Zou B Han J Wang Y Liu R Zhao S Feng L . Semi-structural interview-based chinese multimodal depression corpus towards automatic preliminary screening of depressive disorders. IEEE Trans Affect Computing. (2023) 14:2823–38. doi: 10.1109/TAFFC.2022.3181210

[60] Girard JM Chu WS Jeni LA Cohn JF . “ Sayette Group Formation Task (GFT) Spontaneous Facial Expression Database,” In: 2017 12th IEEE International Conference on Automatic Face & Gesture Recognition (FG 2017), Washington, DC, USA. (2017) 581–8. doi: 10.1109/FG.2017.144, PMID: PMC5876025 29606916

