# Supplementary material for: Three-dimensional facial features of suicide risk in females with depression
Source: Front Psychiatry. 2026 Jan 22;16:1650104. doi: 10.3389/fpsyt.2025.1650104 (PMC12873306; doi:10.3389/fpsyt.2025.1650104)
Supplement: Supplementary file 1 [file Table1.docx]

**Supplemental Content**

**Three-Dimensional Facial Features of Suicide Risk in Females with Depression**

**eMethds**

**eTable 1.** Landmarks of 32 Distance Features

**eTable 2.** Landmarks of 12 Angel Features

**eTable 3.** Landmarks of 2 Area Features

**eMethods**

The Methodology for Calculating Facial Features

A. Distance features

Assuming two points are $P_{A} = \left( x_{1}, y_{1}, z_{1} \right)$ and $P_{B}=\left( x_{2},y_{2},z_{2} \right)$, then their projection distances on the are $D_{x}=\left| x_{1}-x_{2} \right|$, $D_{y}=\left| y_{1}-y_{2} \right|$ and $D_{z}=\left| z_{1} - z_{2} \right|$ respectively. Then, the Euclidean distance is calculated as:

$$Euc. Dist.=\sqrt{\left( \text{D}_{\text{x}} \right)^{2}+\left( \text{D}_{\text{y}} \right)^{2}+\left( \text{D}_{\text{z}} \right)^{2}}$$

The landmarks of 32 distance features are presented in eTable 1 in the supplementary material.

B. Angel features

To calculate the angle between two points, $P_{A}$ and $P_{B}$, in one direction, for example, the x-axis, there will be.

$$theta_{x}=\arccos\left( \frac{x_{1}-x_{2}}{\sqrt{\left( x_{1}-x_{2} \right)^{2}}} \right)\text{ rad},\text{ where }\theta_{x}\in\left[ 0,\pi\right]$$

Similarly, $\theta_{y}$ and $\theta_{z}$ can be represented in this way. The landmarks of the 12 angel features are presented in eTable 2 in the supplementary material.

C. Area features

The calculation method of the area of a polygon is also based on its vertex coordinates. Given a polygon represented as a list of vertices $\{\left( x_{i},y_{i} \right)\}$, where$i = 1, 2, \ldots, n$, a variant of the Shoelace formula is employed to compute the area of the polygon, iterating over each pair of consecutive vertices to calculates the signed area of each trapezoid formed by these consecutive vertices. The signed area is given by:

$$Area=\sum_{i=1}^{n} \left( x_{i}\cdot y_{i+1}-x_{i+1}\cdot y_{i} \right)$$

Finally, the computed area is divided by 2 to obtain the absolute value of the polygon's area. The landmarks of 2 area features are presented in Table 3 in the supplementary material.

Upon computation, we ultimately obtained 32 distance features, 12 angle features, and 2 area features based on the abovementioned three algorithms. One extra feature (Forehead-height midpoint projection) involves taking the average of the z-coordinate values of point 23 and point 28.

**eTable 1.** **Landmarks of 32 Distance Features**

|  | Landmark 1 | Landmark 2 |
| --- | --- | --- |
| projection onto the x-axis |  |  |
| Face width | 55 | 71 |
| Part Leye x | 9 | 12 |
| Part Reye x | 15 | 18 |
| Part nose x | 4 | 8 |
| Dist Leye nose | 0 | 12 |
| Dist Reye nose | 0 | 15 |
| Part mouth x | 31 | 37 |
| Jaw width | 58 | 68 |
| projection onto the y-axis |  |  |
| Face length | 26 | 53 |
| Philtrum length flat | 6 | 34 |
| Part Leyepit y | 13 | 24 |
| Part Reyepit y | 20 | 27 |
| Part Leye y | 10 | 14 |
| Part Reye y | 16 | 20 |
| Part nose y | 0 | 6 |
| projection onto the z-axis |  |  |
| Nose height | 3 | 6 |
| Nose depth | 0 | 6 |
| Philtrum depth | 6 | 34 |
| Chin depth | 46 | 63 |
| Euclidean distance |  |  |
| Forehead width | 21 | 30 |
| Left eye width | 9 | 12 |
| Right eye width | 15 | 18 |
| Dist inner eyes | 12 | 15 |
| Dist outer eyes | 9 | 18 |
| Nose width | 4 | 8 |
| Nose length | 0 | 3 |
| Philtrum length | 6 | 34 |
| Dist nose lip | 6 | 40 |
| Upper lip thick | 34 | 40 |
| Lower lip thick | 46 | 52 |
| Mouth width | 31 | 37 |
| Chin length | 46 | 63 |

**eTable 2. Landmarks of 12 Angel Features**

|  | Landmark 1 | Landmark 2 |
| --- | --- | --- |
| Angle in the x-axis direction |  |  |
| Left eye slope x | 9 | 12 |
| Right eye slope x | 15 | 18 |
| Philtrum slope x | 6 | 34 |
| Chin slope x | 63 | 46 |
| Angle in the y-axis direction |  |  |
| Left eye slope y | 9 | 12 |
| Right eye slope y | 15 | 18 |
| Philtrum slope y | 6 | 34 |
| Chin slope y | 63 | 46 |
| Angle in the z-axis direction |  |  |
| Left eye slope z | 9 | 12 |
| Right eye slope z | 15 | 18 |
| Philtrum slope z | 6 | 34 |
| Chin slope z | 63 | 46 |

**eTable 3. Landmarks of 2 Area Features**

|  | Landmarks |
| --- | --- |
| The absolute value of the polygon's area |  |
| Left eye area | 15,16,17,18,19,20 |
| Right eye area | 9,10,11,12,13,14 |
